# Low-energy small language models with retrieval-augmented generation can surpass large-model performance in rheumatology

**DOI:** 10.3389/fmed.2026.1817215

**Published:** 2026-05-08

**Authors:** Sabine Felde, Rüdiger Buchkremer, Gamal Chehab, Christian Thielscher, Jörg H. W. Distler, Matthias Schneider, Jutta G. Richter

**Affiliations:** 1 Institute of IT Management and Digitization Research (IFID), FOM University of Applied Sciences, Düsseldorf, Germany; 2 Department of Rheumatology, University Hospital Düsseldorf, Medical Faculty of Heinrich-Heine-University, Düsseldorf, Germany; 3 Hiller Research Center, Medical Faculty of Heinrich-Heine-University Düsseldorf, Düsseldorf, Germany; 4 Department of Rheumatology and Clinical Immunology, KEM Kliniken Essen-Mitte, Essen, Germany; 5 Competence Center for Medical Economics, FOM University of Applied Sciences, Essen, Germany

**Keywords:** AI-based clinical decision support, artificial intelligence, retrieval-augmented generation, rheumatology, small language models

## Abstract

**Background:**

Large language models (LLMs) are increasingly explored for clinical decision support but are limited by high computational and energy demands. Smaller language models (SLMs), particularly when combined with retrieval-augmented generation (RAG), may offer a more sustainable alternative. Rheumatology, characterized by diagnostic complexity and guideline-driven management, represents a suitable test domain.

**Methods:**

Five state-of-the-art language models (GPT-4o, Mixtral-8 × 7b-32768, Llama-3.1-Nemotron-70b-Instruct, Qwen-Turbo 2.5, Claude-3.5-Sonnet) were evaluated regarding their suitability for clinical decision support using ten standardized, anonymized rheumatology cases. Models were assessed with and without RAG, and with or without a predefined diagnosis. Diagnostic and therapeutic accuracy were quantified using F1 scores. Factual consistency and relevance were assessed using the Retrieval-Augmented Generation Assessment Score (RAGAS).

**Results:**

Mixtral-8 × 7b-32768 with RAG achieved the highest diagnostic (72%) and therapeutic (73%) F1 scores. Nemotron-70b showed strong diagnostic performance without RAG (71%), while Qwen-Turbo performed well in therapeutic recommendations without retrieval (72%). The highest RAGAS score was observed for Mixtral with RAG (81%). Performance regarding clinical decision support varied substantially across models and configurations.

**Conclusion:**

SLMs combined with RAG can match or exceed the performance of larger LLMs for clinical decision support while requiring significantly fewer computational resources. Despite promising results, clinically relevant errors persisted across all models, underscoring the need for expert oversight and further real-world validation.

## Introduction

Artificial intelligence (AI), and particularly large language models (LLMs), has expanded rapidly across clinical contexts during recent years. Their capacity to synthesize large volumes of unstructured text, identify patterns and produce context-specific, guideline-aligned recommendations has made them a promising tool for decision support in complex clinical environments (1, 2). Despite this potential, the introduction of LLMs into routine care is constrained by extensive computational and energy requirements and by the need for substantial technical infrastructure, making their integration challenging for many healthcare facilities (2, 3).

Smaller language models (SLMs) represent a possible solution. Defined as models with fewer than 100 billion parameters, SLMs require substantially fewer resources for inference and can often be deployed on local hospital hardware. This is of particular relevance in contexts where data security, latency constraints, legal frameworks or staffing limitations demand local hosting rather than cloud-based solutions (3, 4).

Rheumatology is a domain where diagnostic uncertainty is common due to overlapping symptoms across inflammatory, autoimmune, and degenerative conditions. Inflammatory rheumatic diseases, including rheumatoid arthritis (RA), axial spondyloarthritis (axSpA), and connective tissue diseases, are frequently associated with substantial diagnostic delays, often ranging from several months to multiple years (e.g., in axSpA more > 5 years) depending on the disease entity (5–7). For RA in Germany the diagnostic delay exceeds 14 months, driven by specialist shortages and long waiting times (3). Recent studies show that patients often perceive AI-generated responses as comparable to physician responses, although experts identify substantial inaccuracies and potential for clinically relevant errors (8–11). These observations underline the need for careful evaluation of AI systems in this field.

Retrieval-augmented generation (RAG) has been proposed as a method to mitigate several limitations of LLMs and SLMs, including hallucinations, outdated training knowledge and inconsistent guideline adherence (12, 13). Through automated retrieval of external documents—such as clinical guidelines—RAG enables the model to integrate accurate, up-to-date information at inference time. While RAG has shown promising results in general medical applications, its use in rheumatology has only recently begun to be explored (14). In particular, Madrid-Garcia et al. recently described a comparable RAG-based framework in rheumatology that integrated EULAR and ACR recommendations and significantly improved the factual accuracy, safety, and completeness of LLM-generated responses compared with a baseline model, thereby supporting the use of retrieval-based architectures for clinically grounded decision support (15).

We conducted a comparative evaluation of five advanced LLMs and SLMs, with and without RAG, regarding their suitability for clinical decision support across diagnostic and therapeutic tasks using standardized rheumatology cases. The aim was to determine whether SLMs, particularly when augmented with RAG, could achieve clinically relevant performance while remaining computationally feasible for real-world clinical deployment.

## Materials and methods

### Study design and patient data

This study used ten standardized patient cases derived from anonymized records of individuals treated at a rheumatology tertiary center in Germany. Cases included diagnoses, laboratory medications, disease course and treatment decisions. All material was fully anonymized and reformatted to ensure a consistent structure. No identifiable patient information was included.

### Evaluated models

Five state-of-the-art language models were selected based on their performance in public leaderboards (16) and their architectural diversity, including both dense and mixture-of-experts (MoE) architectures (17):

GPT-4o (OpenAI);
Mixtral-8 × 7b-32768 (Mistral AI);
Llama-3.1-Nemotron-70b-Instruct (Nvidia);
Qwen-Turbo 2.5 (Alibaba Cloud);
Claude-3.5-Sonnet (Anthropic).

In accordance with recent literature, SLMs were defined as models with fewer than 100 billion parameters (3, 4). Models such as Nemotron-70B, while technically falling below this threshold, represent medium-sized architectures and should therefore be interpreted separately from substantially smaller models such as Mixtral-8 × 7b. However, compared to a large language model (LLM) with a size on the order of trillions, 7B can still be considered small.

### Retrieval-augmented generation pipeline

The RAG pipeline was implemented using a pragmatic and computationally efficient retrieval architecture. Established rheumatology guidelines, including those from the European Alliance of Associations for Rheumatology (EULAR), the American College of Rheumatology (ACR), and the Scottish Intercollegiate Guidelines Network (SIGN), were used as the external knowledge base. These guideline documents were indexed using FAISS (IndexFlatL2) (12), which was selected because of its simplicity, deterministic behavior, and suitability for small to medium-sized clinical document collections without requiring additional index training.

Text representations were generated using the Sentence-BERT model distilbert-base-nli-stsb-mean-tokens (13). This embedding model was chosen because it provides efficient sentence-level semantic representations and has been widely applied in semantic similarity and biomedical text retrieval tasks, offering a practical balance between retrieval quality and computational efficiency.

For retrieval and reranking, the guideline documents were segmented into paragraph-based text passages using semantically coherent sections separated by empty lines. This chunking approach was selected to preserve contextual coherence while maintaining low implementation complexity. For each query, the top three most relevant passages were retrieved (top-k = 3) and subsequently reranked using FlashRank (ms-marco-MiniLM-L-12-v2) (14) to improve the relevance ordering of the retrieved guideline excerpts before they were provided to the models during inference.

To maintain manageable context windows and ensure consistent input across models, retrieved passages were truncated to a maximum context length of approximately 1,000 words before inference.

### Evaluation conditions

Each model was tested under four conditions: (1) Without RAG and without a predefined diagnosis; (2) With RAG and without a predefined diagnosis; (3) Without RAG and with a predefined diagnosis; (4) With RAG and with a predefined diagnosis.

Standardized prompts used few-shot and chain-of-thought prompting techniques (18, 19) Representative prompt examples are provided in the Supplementary materials. Prompt design was guided by the aim to ensure consistent diagnostic reasoning and structured therapeutic recommendations across all tested models.

Responses were independently assessed by two senior rheumatologists across all three evaluation dimensions. To evaluate rating consistency, inter-rater agreement was quantified using Cohen’s kappa coefficient and exact percentage agreement.

### Outcome measures

Diagnostic and therapeutic performance was quantified using the F1 score, which reflects the harmonic mean of precision and recall and thus provides a balanced measure of classification accuracy, particularly in settings with class imbalance (20). Factual consistency and relevance were evaluated using the Retrieval-Augmented Generation Assessment Score (RAGAS) (21, 22). Ground truth was established based on evidence-based guidelines (23).

## Results

### Overall model performance

Substantial variation was observed across models and testing configurations. Retrieval-augmented generation (RAG) generally improved diagnostic and therapeutic accuracy, although the magnitude of improvement varied considerably depending on model architecture (Table 1). Small language models (SLMs) benefited most consistently from RAG, whereas larger models demonstrated higher baseline factual performance but less incremental gain.

**TABLE 1:** Model performance summary - top F1 and Retrieval-Augmented Generation Assessment Scores (RAGAS) scores of evaluated models.

Model	Model size	Configuration	Metric	Top score (%)	Evaluation assessment
Mixtral-8 × 7b-32768	SLM	RAG, no pre-diagnosis	F1-Dx	72	Best diagnostic performance with RAG
Mixtral-8 × 7b-32768	SLM	RAG, no pre-diagnosis	F1-Tx	73	Best treatment recommendation performance
Nemotron 70b instruct	SLM	No RAG, no pre-diagnosis	F1-Dx	71	Strong diagnostic performance without RAG
Qwen-Turbo	SLM	No RAG, no pre-diagnosis	F1-Tx	72	Strong treatment performance without RAG
GPT-4o	LLM	RAG, with pre-diagnosis	RAGAS	74	Good factual alignment with external knowledge
Mixtral-8 × 7b-32768	SLM	RAG, with pre-diagnosis	RAGAS	81	Highest RAGAS score
Claude-3.5-Sonnet	LLM	No RAG, with pre-diagnosis	RAGAS	80	High RAGAS score without RAG integration

This table summarizes the highest diagnostic and therapeutic F1 scores and RAGAS scores achieved by each model under the tested conditions. F1-Dx, F1 score for diagnosis; F1-Tx, F1 score for treatment recommendation. RAG, retrieval-augmented generation: retrieving relevant documents before generating an answer. Scores represent the highest achieved in the respective category across test conditions.

### Diagnostic accuracy

Mixtral-8 × 7b-32768 with RAG achieved the highest diagnostic F1 score (72%), outperforming all large and small models (Table 1). A comprehensive overview of all results is provided in the appendix. Nemotron-70b-Instruct achieved an F1 score of 71% without RAG, suggesting a strong internal representation of rheumatological knowledge, while GPT-4o demonstrated moderate and stable performance across configurations.

### Therapeutic recommendation accuracy

Therapeutic performance followed similar patterns. Mixtral-8 × 7b-32768 with RAG achieved the highest F1 score (73%) (Table 1). Qwen-Turbo 2.5 without RAG performed nearly as well (F1 = 72%), indicating that the model encodes useful therapeutic guidance internally, even in the absence of retrieval support.

### RAGAS performance

**FIGURE 1:**
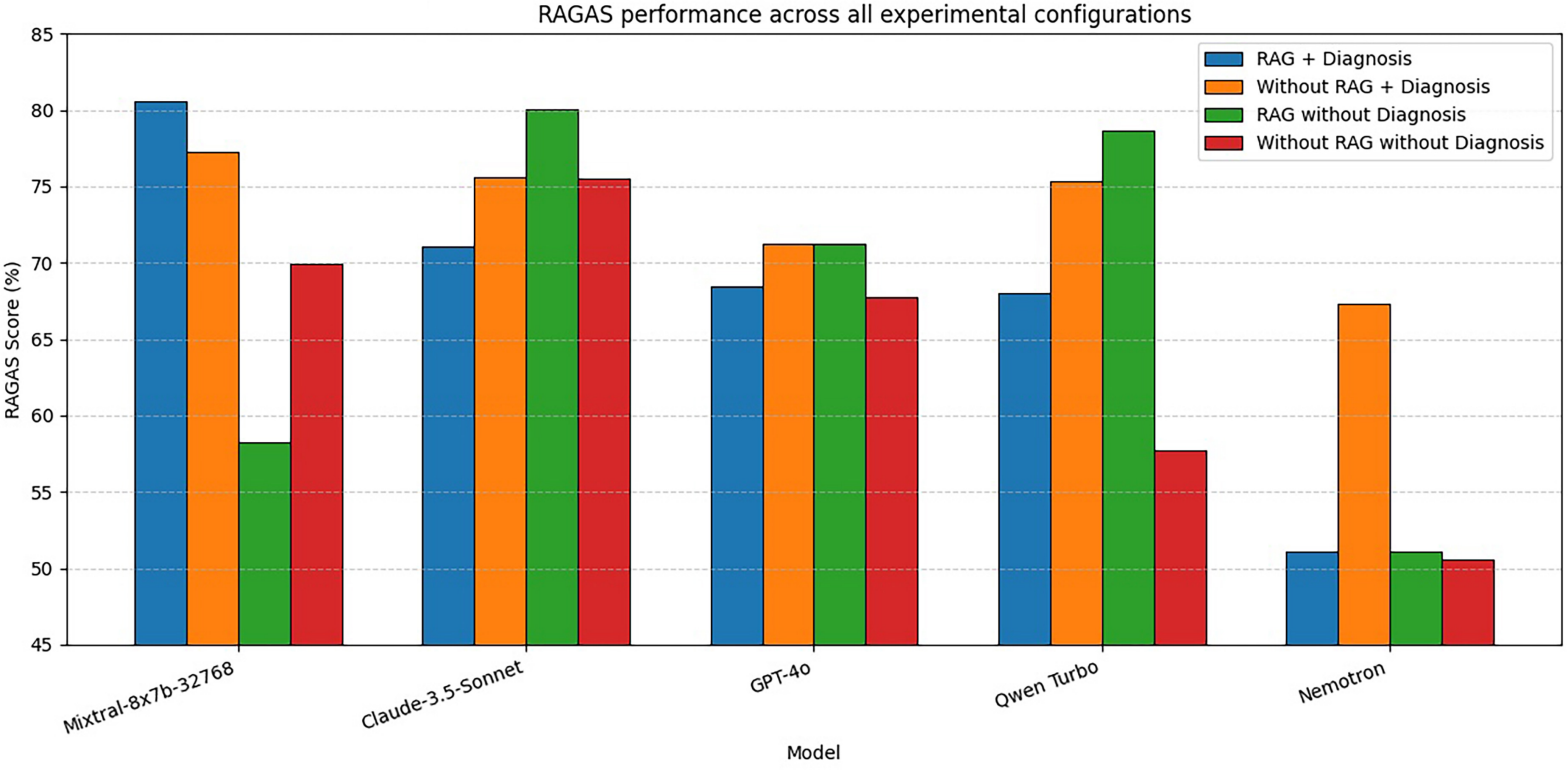
Retrieval-Augmented Generation Assessment Scores (RAGAS) performance across all experimental configurations. RAGAS of all models under four testing conditions. Mixtral-8 × 7b-32768 with RAG and Claude-3.5-Sonnet without RAG demonstrated the highest factual alignment. Nemotron exhibited substantial variability across conditions.

The RAGAS evaluation revealed distinct differences in factual alignment and hallucination resilience across architectures (Figure 1). Mixtral-8 × 7b-32768 with RAG achieved the highest overall RAGAS score (81%), while Claude-3.5-Sonnet without RAG demonstrated excellent intrinsic alignment (RAGAS 80%), suggesting a robust internal medical corpus. Nemotron-70b showed the greatest variability (RAGAS 51%–67%), indicating substantial sensitivity to retrieval quality.

However, expert review by the rheumatologists showed that 9% of generated responses were not suitable for clinical use. This was due, for example, to unsafe or hallucinated therapeutic recommendations—i.e., recommendations based on prior treatments that were assumed by the model but had not actually been administered to the patient. Across all three evaluation dimensions, the inter-rater agreement between the two senior rheumatologists was substantial (Cohen’s κ = 0.47), with an agreement rate of 73%. These findings highlight that strong quantitative scores do not fully capture clinically relevant errors.

## Discussion

This study provides a comparative evaluation of five advanced language models for clinical decision support in Rheumatology. The findings show that SLMs, particularly Mixtral-8 × 7b-32768, can achieve diagnostic and therapeutic performance equal to or better than that of large frontier LLMs when combined with RAG. These results challenge the assumption that larger parameter counts necessarily lead to improved clinical reasoning (1, 2, 11, 24, 25). This finding complements recent work by Madrid-Garcia et al., who likewise demonstrated the feasibility of retrieval-augmented generation in rheumatology using a similar guideline-supported approach (15). While their study primarily established the applicability of RAG in this field, our results extend these observations by directly comparing multiple large and small language model architectures under different experimental conditions and by specifically addressing the question of whether smaller, more resource-efficient models may represent a sustainable alternative for clinical deployment and implementation.

The consistent improvement observed with RAG underscores its potential role in mitigating hallucinations and integrating current guidelines during inference (12, 14). However, the degree of benefit varied by architecture. Models with strong internal knowledge, such as Nemotron and Claude-3.5-Sonnet, showed less dependence on retrieval.

From a clinical perspective, even small deviations in accuracy metrics may mask errors with meaningful clinical consequences. Oversights involving contraindications, comorbidities or red-flag symptoms may lead to harmful recommendations, even when overall F1 scores appear favorable (26). As a result, none of the evaluated models should be used without expert supervision.

The practical advantages of SLMs are substantial. Their reduced computational requirements, lower energy consumption and potential for local deployment make them suitable for healthcare systems with limited digital infrastructures (3, 4). In Rheumatology, where diagnostic delays remain a challenge and specialist shortages persist (3), SLMs may help streamline triage, summarize documentation and accelerate initial assessment (27).

The study has several limitations. The sample size was limited to ten cases, which constrains generalizability and limits conclusions regarding the behavior of the language models across other clinical scenarios and diagnoses. Furthermore, due to the sample size no confidence intervals, formal hypothesis testing, or inter-rater agreement metrics were calculated, which limits the interpretation of whether observed differences between models are statistically meaningful. Standardized prompt engineering may not fully reflect real-world clinical usage. Metrics such as F1 and RAGAS, while useful for quantitative comparison, do not fully capture clinical nuance. In addition, the performance of the RAG pipeline may depend on methodological design choices such as embedding model selection, chunking strategy, reranking configuration, and retrieval depth. Thus, larger, prospective evaluations in real-world clinical settings are needed (11, 14, 23, 28).

In conclusion, SLMs combined with RAG provide a promising and more accessible approach to clinical decision support in rheumatology. They offer performance comparable to or exceeding that of computationally expensive LLMs, while remaining feasible for deployment in diverse healthcare environments. Expert review by the rheumatologists showed that 9% of responses were not suitable for clinical use. The persistence of clinically relevant errors underscores the need for caution and ongoing validation.

## Data Availability

The raw data supporting the conclusions of this article will be made available by the authors, without undue reservation.
