# Supplementary material for: Low-energy small language models with retrieval-augmented generation can surpass large-model performance in rheumatology
Source: Front Med (Lausanne). 2026 May 8;13:1817215. doi: 10.3389/fmed.2026.1817215 (PMC13193909; doi:10.3389/fmed.2026.1817215)
Supplement: Supplementary file 1 [file Data_Sheet_1.pdf]

Prompt Template 1

Combined Diagnostic + Therapeutic Reasoning

No RAG / No Predefined Diagnosis

System prompt:
You are an expert rheumatology clinical decision support assistant.

Use structured clinical reasoning and provide evidence-based recommendations.

Few-shot example:
Example case:
Patient with symmetric polyarthritis, positive RF and anti-CCP antibodies.

Expected output:
Diagnosis: Rheumatoid arthritis
Treatment: Methotrexate 15 mg/week + folic acid

Task prompt:
Please reason step by step:

analyse symptoms

analyse laboratory findings

evaluate differential diagnoses

derive final diagnosis

propose treatment

Patient case:
[Insert standardized anonymized patient case]

Prompt Template 2

Combined RAG + Chain-of-Thought + Few-shot

No Predefined Diagnosis

System prompt:
You are an expert rheumatology clinical decision support assistant.

Use both the patient information and the provided guideline excerpts.

Few-shot example:
Example case:
Patient with inflammatory back pain and HLA-B27 positivity.

Expected output:
Diagnosis: Axial spondyloarthritis
Treatment: NSAIDs, MRI sacroiliac joints

Task prompt:
Please reason step by step:

analyse symptoms

analyse laboratory findings

compare with guideline excerpts

evaluate differential diagnoses

derive final diagnosis

recommend treatment

Patient case:
[Insert standardized anonymized patient case]

Retrieved guideline excerpts:
[Insert top-3 retrieved guideline passages]

Prompt Template 3

Combined Therapy Planning Prompt

Predefined Diagnosis + RAG

System prompt:
You are an expert rheumatology clinical decision support assistant.

The diagnosis has already been established.

Use the patient case and the guideline excerpts to provide an evidence-based treatment plan.

Please reason step by step:

assess disease activity

evaluate contraindications

assess comorbidities

propose first-line treatment

propose escalation strategy

recommend monitoring

Confirmed diagnosis:
[Insert diagnosis]

Patient case:
[Insert standardized anonymized patient case]

Retrieved guideline excerpts:
[Insert top-3 retrieved guideline passages]

Prompt Template 4

Combined Safety-focused Clinical Decision Support Prompt

System prompt:
You are an expert rheumatology clinical decision support assistant.

Pay special attention to:

contraindications

red-flag symptoms

comorbidities

medication risks

Please provide:

diagnosis

treatment

risks

follow-up recommendations

clinical justification

Patient case:
[Insert standardized anonymized patient case]

Retrieved guideline excerpts:
[Insert top-3 retrieved guideline passages]

Model performance - F1 and RAGAS scores of all evaluated models

Model	Model Size	Configuration	F1-Dx (%)	F1-Tx (%)	RAGAS (%)
Mixtral-8x7b-32768	SLM	RAG + Diagnosis	69.7	66.1	80.61
Mixtral-8x7b-32768	SLM	Without RAG + Diagnosis	64.7	61.1	77.30
Mixtral-8x7b-32768	SLM	RAG without Diagnosis	71.8	73.0	58.23
Mixtral-8x7b-32768	SLM	Without RAG without Diagnosis	63.3	66.1	69.92
Claude-3.5-Sonnet	LLM	RAG + Diagnosis	58.9	62.0	71.05
Claude-3.5-Sonnet	LLM	Without RAG + Diagnosis	64.3	60.1	75.57
Claude-3.5-Sonnet	LLM	RAG without Diagnosis	62.6	65.7	80.02
Claude-3.5-Sonnet	LLM	Without RAG without Diagnosis	49.5	67.8	75.52
GPT-4o	LLM	RAG + Diagnosis	60.5	61.9	68.45
GPT-4o	LLM	Without RAG + Diagnosis	60.7	62.8	71.25
GPT-4o	LLM	RAG without Diagnosis	66.2	71.2	71.25
GPT-4o	LLM	Without RAG without Diagnosis	64.8	66.5	67.79
Qwen Turbo	SLM	RAG + Diagnosis	60.0	60.3	67.99
Qwen Turbo	SLM	Without RAG + Diagnosis	58.4	58.1	75.38
Qwen Turbo	SLM	RAG without Diagnosis	66.8	72.3	78.63
Qwen Turbo	SLM	Without RAG without Diagnosis	63.4	70.8	57.71
Nemotron	SLM	RAG + Diagnosis	60.3	55.2	51.08
Nemotron	SLM	Without RAG + Diagnosis	66.1	59.7	67.33
Nemotron	SLM	RAG without Diagnosis	63.9	63.6	51.13
Nemotron	SLM	Without RAG without Diagnosis	70.9	63.5	50.60
